# Association of Daily Dietary Intake and Inflammation Induced by Marathon Race

**DOI:** 10.1155/2019/1537274

**Published:** 2019-10-07

**Authors:** Bharbara N. Passos, Mirthes C. Lima, Ana P. R. Sierra, Rodrigo A. Oliveira, Jaqueline F. S. Maciel, Richelieau Manoel, Julliane I. Rogante, João B. Pesquero, Maria F. Cury-Boaventura

**Affiliations:** ^1^Institute of Physical Activity and Sports Sciences, Cruzeiro do Sul University, Sao Paulo 01506-000, Brazil; ^2^School of Physical Education and Sport, University of São Paulo, Sao Paulo 05508-030, Brazil; ^3^Department of Biophysics, Federal University of Sao Paulo, Sao Paulo 04023-062, Brazil

## Abstract

Daily food intake is crucial to maintain health and determine endogenous fuel to practice endurance exercise. We investigated the association between quantity of macronutrient and micronutrient daily intake and inflammation induced by long-distance exercise. *Methods*. Forty-four Brazilian male amateurs' marathon finishers from 30 to 55 years old participated in this study. Blood samples were collected 1 day before, immediately after, and 1 day and 3 days after São Paulo International Marathon. The serum levels of IL-6, IL-1*β*, IL-10, IL-8, IL-12p70, and TNF-*α* were measured to evaluate inflammation. Dietary intake was determined using a prospective method of three food records in the week before marathon race. *Results*. Marathon race promoted an elevation on IL-6, IL-8, IL-1-*β*, and IL-10 immediately after the race. The energy intake (EI), carbohydrate, fiber, folic acid, vitamin E, vitamin D, calcium, magnesium, and potassium intakes was below recommended. Immediately after the marathon race, we observed a negative correlation between IL-8 and daily EI, carbohydrate, fiber, fat, iron, calcium, potassium, and sodium intakes, and higher levels of IL-8 on runners with <3 g/kg/day of carbohydrate intake compared to runners with >5 g/kg/day. We demonstrated a positive correlation between daily carbohydrate intake and IL-10 and a negative correlation between TNF-*α* and % of energy intake recommended, carbohydrate and fiber intakes. Finally, runners with adequate EI had lower levels of IL-1*β* and TNF-*α* compared with low EI immediately after the race. *Conclusion*. Nutrition strategies to promote balanced diet in amateur runners seem to be as important as immunonutrition sports market. Daily food intake, mainly EI, electrolyte and carbohydrate intakes, may modulate exacerbated inflammation after endurance exercise.

## 1. Introduction

Prolonged exercise is a physiological stress which promotes hyperinflammation following anti-inflammatory compensatory response characterized by the increase on inflammatory mediators such as IL-6, IL-8, IL-ra, IL-10, and C-reactive protein (CRP) [1–5]. The exacerbated inflammation contributes to transient immunosuppression, as well as hematological and cardiopulmonary changes, and muscle and renal damage induced by exercise [6, 7].

Nutrients are necessary to provide energy and optimize performance and recovery, maintaining immune health [8–10]. The majority of studies in sports nutrition field have been investigating the impact of nutritional supplements to improve performance, body composition, and immune function. Nowadays, the immunonutrients recommended to improve immune health include high carbohydrate, fruit and vegetable rich in polyphenol intake. The proposed benefits of nutritional components such as probiotics, prebiotics, fish oil, bovine colostrum, vitamins, and minerals to exercise-immune function remain unclear and need further investigation [8, 9].

However, there are few human *in vivo* studies evaluating the quality or quantity of food intake on immune function in physiologically stressed athletes. Chronic low energy availability has been demonstrated to be one of the main factors related to endocrine, metabolic, and immune dysfunction [11, 12]. Recently, studies have been reporting an inadequate daily intake characterized by a low intake of carbohydrate, dietary fiber sources, fruits, dairy beverages, and vegetables in long-distance runners [13–17]. Nutritional strategies that provide energy demands and sufficient specific macro- and micronutrients to support the immune function could avoid consumption of “immune boosting” supplements. Endurance athletes usually make many nutritional mistakes, not based on metabolic scientific evidence, mainly regarding which type of athlete would benefit, or the dose and timing [18].

Daily food intake is crucial to determine endogenous fuel to practice endurance exercise and consequently to maintain immune health. The aim of the present study was to investigate the association between quantity of macronutrient and micronutrient daily intake and inflammation induced by long-distance exercise. We hypnotized that inadequate energy and some nutrient daily intake, such as carbohydrate, could impair hyperinflammatory state after endurance exercise.

## 2. Materials and Methods

### 2.1. Subjects

Forty-four Brazilian male amateurs' marathon finishers from 30 to 55 years old participated in this study. Volunteers were recruited by e-mail provided by São Paulo International Marathon Organization (9^th^ April, 2017). The exclusion criteria included the use of medication to cardiac, metabolic, pulmonary, or kidney injury and the use of alcohol or any kind of drugs and pathologies including systemic arterial hypertension, liver, kidney, metabolic, inflammatory, or neoplastic diseases. After screening history and clinical medical evaluation, subjects were informed of the experimental procedures and possible risks and signed a term of informed consent, which was approved by the Ethics Committee of Dante Pazzanese Institute of Cardiology, Brazil (permit number: 979/2010), in accordance with the Declaration of Helsinki. Measurements of total body mass (kg), height (m), and Body Mass Index (BMI, kg/m^2^) were conducted 1 day before marathon race according to the International Society for the Advancement of Kinanthropometry and expressed as the mean ± SEM. Blood samples (30 mL) were collected in vacuum tubes containing an anticoagulant (0.004% EDTA) 1 day before, immediately after, and 1 day and 3 days after São Paulo International Marathon. Serum samples were isolated by centrifugation (400g, 10 minutes) and stored at -80°C for later cytokine analysis at Cruzeiro do Sul University.

### 2.2. Marathon Race

The marathon race began at 07:30 a.m., and fluid ingestion was allowed *ad libitum* during the race. Water was provided every 2 to 3 km on the running course; sports drinks on 12 km, 21.7 km, 33 km, and 42 km; and potato on 28.8 km. The runners were instructed to consume between 30 and 60 g of carbohydrate per hour and 500 to 1000 mL of water. The weather parameters at São Paulo International Marathon in 2017 between 7 a.m. and 2 p.m. were average temperature of 19.8°C, maximum temperature of 22.6°C, and minimum temperature of 16.7°C, with average relative humidity of 72.8%, maximum relative humidity of 86%, and minimum relative humidity of 61% (National Institute of Meteorology, Ministry of Agriculture, Livestock, and Supply).

### 2.3. Biochemical Parameters

The serum levels of IL-6, IL-1*β*, IL-10, IL-8, IL-12p70, and TNF-*α* were determined using the BD™ Human Inflammatory Cytokine Cytometric Bead Array Kit and BD Accuri cytometer according to the manufacturer's instructions (BD Biosciences, San Jose, CA, USA). The detection limit was 3.6 pg/mL for IL-8, 7.2 pg/mL for IL-1*β*, 2.6 pg/mL for IL-6, 3.3 pg/mL for IL-10, 3.7 pg/mL for TNF-*α*, and 1.9 pg/mL for IL-12p70.

### 2.4. Dietary Intake

Dietary intake was determined using a prospective method of three food records in the week before the marathon race (3^rd^ to 8^th^ April). The runners were oriented to describe all food and drinks that are consumed, meal time, portion size, and food brand, when applicable, two days of week and one day of weekend. The food records were revised by a trained nutrition undergraduate student to check or complete the record one day after the marathon. Dietary intake was analyzed by professional dietbox (http:/dietbox.me) website/app to determine energy intake, macronutrients and micronutrients. The professional dietbox website/app provides nutrients composition from the United States Department of Agriculture-Agricultural Research Service (USDA) food composition database and Brazilian Table of Food Composition database (TACO, University of Campinas, São Paulo, SP, Brazil).

### 2.5. Statistical Analyses

Statistical analyses were performed using the Statistical Package for the Social Sciences (Graph Prism version 6). The normality of the data distribution was determined by the Kolmogorov-Smirnov test and rejects the normality. Differences between the steps (before, immediately after, 1 day after, and 3 days after the race) were tested for significance with the Friedman test with repeated measures and the Müller-Dunn post test. The nonparametric Spearman correlation was determined between absolute value of cytokines (before, immediately after race, or one day after race) and absolute value of macronutrients and micronutrients. Statistical significance was assumed at *p* value < 0.05. The values are presented as mean ± error mean standard of 44 runners.

## 3. Results

The general and training characteristics of all marathon runners are summarized as follows: age, 41.5 ± 1.1 years; weight, 74.4 ± 1.6 kg; height, 1.73 ± 0.0 m; BMI, 24.8 ± 0.4 kg/m^2^; percentage of fat mass, 21.6 ± 0.7%; race time, 267 ± 7 minutes; training experience, 6 ± 0.5 years; time on 10 km race, 46 ± 0.7 minutes; frequency of training, 4.4 ± 0.7 times/week; and training volume, 56 ± 2.1 km/week.

### 3.1. Cytokines

**Figure 1:**
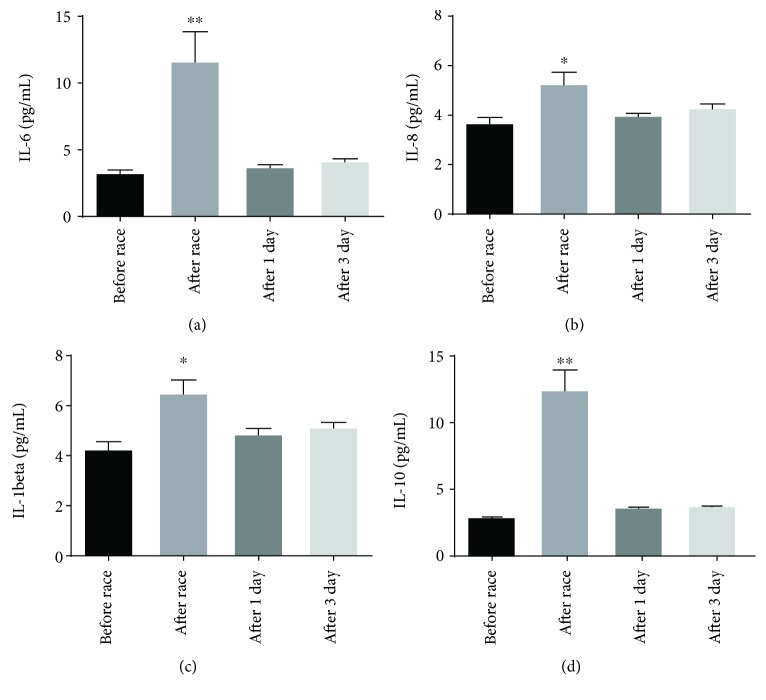
Effect of marathon on cytokines. The inflammatory mediators evaluated were interleukin- (IL-) 6, IL-8, IL-1*β*, and IL-10 before, immediately after, 1 and 3 days after the race. The values are shown as mean ± SEM of 44 runners. ^∗^*p* < 0.01 vs. before race. ^∗∗^*p* < 0.0001 vs. before race.

Marathon race promoted an elevation on IL-6 (by 40%) (Figure 1(a)), IL-8 (by 40%) (Figure 1(b)), IL-1-*β* (by 3.5-fold) (Figure 1(c)), and IL-10 (by 4.1-fold) (Figure 1(d)) serum levels, returning to basal levels one day after the race (Figure 1). We did not observe changes on TNF-*α* and IL-12p70 after the race (data not shown).

### 3.2. Dietary Intake: Macronutrients

The distribution of energy from carbohydrate was lower (48.9 ± 9%) compared to 50 to 60% recommended. The EI and carbohydrate consumption were as 33% and 52% lower than that recommended by the Dietary Reference Intake (2002) and ISSN (2010), respectively (Table 1). The EI was 39.3 ± 2.1 kcal · kg^−1^ FFM·day^−1^ classified as a low energy intake (<45 kcal · kg^−1^ FFM·day^−1^). The cholesterol intake (305.4 ± 160 mg/day), percentage of total fat and protein (19.4%) and sucrose, was close to recommended, and fiber intake (21 ± 2 g/day) consumption was below recommended (DRI, 2002) (Table 1).

### 3.3. Dietary Intake: Micronutrients

The folic acid, vitamin E, and vitamin D intakes were below of DRI recommendation (by 31%, 7%, and 43%, respectively) (Table 2). On the other hand, vitamins A, B1, B2, B3, B6, B12, and C intakes were above reference daily values (by 18%, 38%, 34%, 82%, 81%, 79%, and 61%, respectively) (Table 2).

The daily consumption of iron, phosphorus, manganese, selenium, zinc, or sodium was close or above reference daily values (by 94%, 88%, 10%, 3-fold, 11%, and 73%, respectively), while calcium, magnesium, and potassium were below recommended (by 35%, 33%, and 40%, respectively) (Table 2).

### 3.4. Correlation: Cytokines and Daily Intake

Before the race, IL-6 correlated positively with daily energy intake, macronutrients (carbohydrate, protein, and fat) and vitamin A, B2, calcium, phosphorus, zinc, and potassium (Table 3).

**Figure 2:**
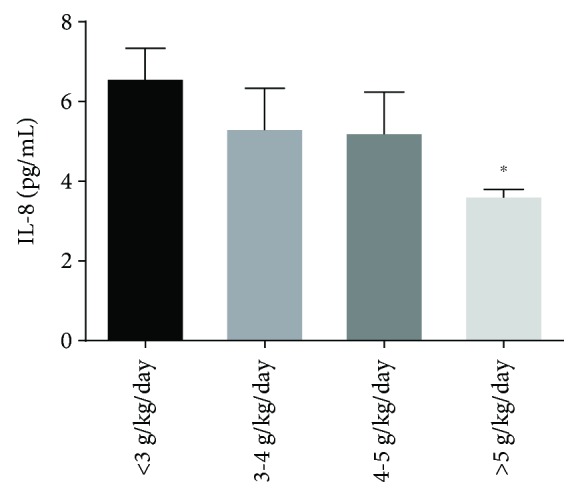
Interleukin- (IL-) 8 concentration of runners with different levels of carbohydrate intake. The runners were divided in 4 groups in accordance with carbohydrate intake: <3 g/kg/day (17 runners), 3-4 g/kg/day (11 runners), 4-5 g/kg/day (8 runners), and >5 g/kg/day (12 runners). The values are shown as mean ± SEM of 8-17 runners. ^#^*p* < 0.01 vs. <3 g/kg/day.

Immediately after the marathon race, we observed a negative correlation between IL-8 and daily energy intake, carbohydrate, fiber, fat, iron, calcium, potassium, and sodium, suggesting that lower intakes are associated with higher IL-8 levels (Table 3). We also demonstrated higher levels of IL-8 on runners with <3 g/kg/day of carbohydrate intake compared to runners with >5 g/kg/day (Figure 2).

One day after the race, we also demonstrated a positive correlation between daily carbohydrate intake and IL-10 (*r* = 0.3, *p* = 0.045), an anti-inflammatory cytokine. Moreover, the proinflammatory cytokine alpha-TNF immediately after the race had a negative correlation with % of energy intake recommended (*r* = 0.31, *p* = 0.04), carbohydrate (*r* = −0.33, *p* = 0.03), and fiber (*r* = −0.38, *p* = 0.01).

**Figure 3:**
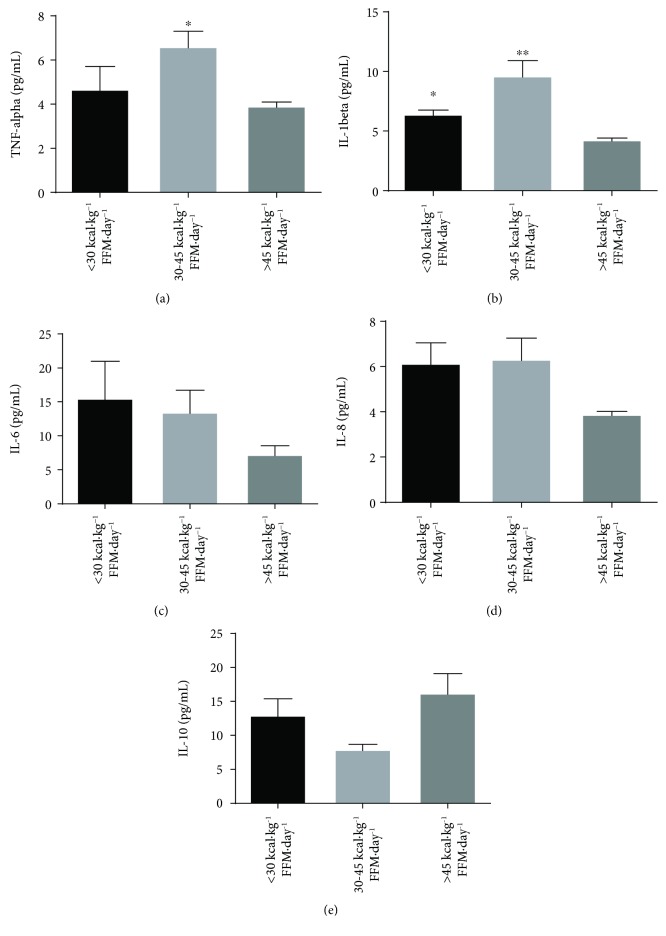
Cytokine release of runners with different levels of energy intake (EI). TNF-*α* (a), IL-1*β* (b), IL-6 (c), IL-8 (d), and IL-10 (e) plasma concentrations of runners within different levels of energy intake were evaluated. The runners were divided in 3 groups in accordance with energy intake: relative energy deficiency, <30 kcal·kg^−1^ FFM·day^−1^ (14 runners); low EI, 30-45 kcal·kg^−1^ FFM·day^−1^ (14 runners); and adequate EI, >45 kcal·kg^−1^ FFM·day^−1^ (16 runners). The values are shown as of mean ± SEM of 8-17 runners. ^∗^*p* < 0.05 vs. <45 kcal·kg^−1^ FFM·day^−1^ and ^∗∗^*p* < 0.0001 vs. <45 kcal·kg^−1^ FFM·day^−1^.

Amateur runners with adequate EI (>45 kcal·kg^−1^ FFM·day^−1^) had lower levels of IL-1*β* compared to low EI (30-45 kcal·kg^−1^ FFM·day^−1^) and relative energy deficiency (<30 kcal·kg^−1^ FFM·day^−1^) immediately after the race and also lower levels of TNF-*α* compared to runners with low EI (30-45 kcal·kg^−1^ FFM·day^−1^) immediately after the race (Figures 3(a) and 3(b)). IL-8 and IL-6 levels also tended to be reduced, and IL-10 tended to be greater in runners with adequate EI immediately after the race (Figures 3(c)–3(e)).

## 4. Discussion

The inflammatory process induced by exercise may be influenced by many factors including food adequate intake [8]. The daily intake of energy, carbohydrate, fiber, vitamins B3, B6, and D, calcium, magnesium, and potassium, was below the daily reference value in amateur's marathon runners in the precompetition period. We suggest that proinflammatory cytokines IL-8 or TNF-alpha release induced by exercise are associated with low energy, carbohydrate, fiber, and/or mineral daily intake. Moreover, higher daily consumption of carbohydrate may improve IL-10 levels in the recovery period of long-distance exercise.

Long-distance runners require higher amount of energy and macronutrients (protein and carbohydrate) and micronutrients (minerals such as iron, magnesium, sodium, and potassium) to support energy expenditure and hydroelectrolytes loss during exercise and to lead the recovery of lean mass, electrolytes, and glycogen store [18, 19]. In this study, we observed a low daily intake of energy, carbohydrate, fiber, vitamins B3, B6, and D, calcium, magnesium, and potassium, in marathon runners. We reported lower EI by 33% and carbohydrate intake by 52%, in accordance with previous studies which reported low energy availability in male athletes [20]. Pugh et al. [17] reported a similar daily energy intake value and daily carbohydrate and protein intake value one day before the race in recreational runners. In spite of dietary intake was assessed by a standard method, food record, with an appropriate database, researchers have reported an underestimate intake in athletes by around 20% for many reasons such as irregular meal patterns and underestimate portion sizes [19]. We suggested that even with the possible interference of method, nutrient deficiency was clear and the runners should be advised to improve the diet, mainly in a precompetition period.

Relative Energy Deficiency in Sport (RED-S) proposed by Mountjoy et al. [12] refers to the 10 physiological and 10 performance-related effects of low EI for male and female athletes [12, 21]. RED-S modulate endocrine physiological response induced by exercise that may affect cortisol levels in response to prolonged exercise but also to starvation and glycogen depletion. RED (<30 kcal·kg^−1^ FFM·day^−1^) accomplished by carbohydrate deficiency also decrease protein synthesis, glucose utilization, mobilization of fat stores, metabolic rate, and production of growth hormone. Cortisol and other hormones such as insulin, IGF-1, modulated by RED-S also could contribute to immune dysfunction [11, 22, 23]. In fact, we observed higher levels of proinflammatory cytokines in runners with relative energy deficiency.

Immune cells need energy fuel substrates, mainly carbohydrate and fat, and micronutrients (zinc, iron, copper, selenium, magnesium, and vitamins A, B6, C, D, and E) to multiple protective enzyme systems involved on immune function [9]. In the 1990s, many researchers demonstrated that ingestion of carbohydrate supplements (30–60 g/h) during endurance/intense exercise attenuated increases in blood neutrophil and monocyte counts, stress hormones, IL-6, IL-10, and IL-1ra and improve immune function. It is also a consensus that utilization of carbohydrate during endurance exercises improve performance and endogenous glycogen stores [18, 24]. Endogenous glycogen stores and circulating plasma glucose are fuel sources for energy provision during endurance exercise, and fatigue coincides with depletion of glycogen store. Carbohydrate intake during exercise seems to suppress endogenous carbohydrate oxidation by around 10% [24]. However, the amount of muscle glycogen oxidation during prolonged exercise is high, around 100 g per hour. The important factor that influences the endogenous glycogen store may be the daily carbohydrate intake, but studies evaluating the role of daily carbohydrate intake levels on immune function in athletes are scarce. In our study, we observed the association between inflammation after the race (IL-8 and TNF-alpha levels) and daily energy, carbohydrate, and fat intakes, and we suggest that lower fuel substrate intake may contribute to fatigue and stress hormone release causing pronounced inflammation. Moreover, the anti-inflammatory cytokine IL-10 was positively associated with carbohydrate intake contributing to understanding of anti-inflammatory effect of carbohydrate after endurance or intense exercise.

The fiber intake also was correlated negatively with inflammation after the race. The main sources of fiber are fruit and vegetable food or juice rich in carbohydrates that seems to be anti-inflammatory and polyphenols which elevate plasma levels of gut-derived phenolics promoting antioxidative and anti-inflammatory capacity [25]. However, the polyphenol intake was not evaluated and the optimal polyphenol intake for athletes has not been defined, and intake recommendations have not been established for humans [9].

Iron balance is essential for health and performance in athletes [26, 27]. Iron deficiency with or without anemia reduces the transport of oxygen and aerobic energy system in the skeletal muscle [28]. In addition, iron regulates many redox reactions crucial for intermediary metabolism [29]. Impaired iron levels by low consumption of iron and inflammation are the major stimulus to hepcidin release from hepatic tissue, and acute-phase protein, which is responsible to inhibit ferroportin expressed in the duodenal enterocyte, hepatocyte, and macrophage intestinal decreasing iron absorption and macrophage iron release from senescent erythrocytes increasing the risk to anemia in athletes [28, 30, 31].

The association between calcium, potassium, and sodium with IL-8 also suggests the importance of daily electrolytic reposition in athletes. During exercise, dehydration occurs due to heat stress and heat load from the thermal energy yield of metabolism. Heat stress induces sweating and subcutaneous vasodilation promoting hypohydration hyperosmotic with loss of electrolytes such as potassium, sodium, and calcium [32, 33]. The dehydration during bouts of training is harmful for physiological adaptation at the intense exercise. The hyponatremia also seems a result of overconsumption of hypotonic fluids and fluid retention, when the athletes release inappropriately arginine vasopressin [34]. The role of hypohydration on inflammation may involve neuroendocrine response, oxidative stress, and muscle metabolism changes but remains unclear [32, 33]. The impairment on the osmolality promotes an increase on ROS and intracellular calcium that activate protease on muscle cells leading systemic inflammation [34].

The IL-6 is an adipomyokine that is released by the muscle after an acute bout of exercise and by adipose tissue or with increased body fat content, with paradoxical autocrine, paracrine, and endocrine effects [11, 35]. Before the race, IL-6 presented a positive correlation with daily energy intake, macronutrients (carbohydrate, protein, and fat) and vitamin A, B2, calcium, phosphorus, zinc, and potassium. Some researchers have been investigating the role of diet intervention on inflammatory markers in overweight and obese people but not in athletes. The restriction of energy seems to reduce inflammatory mediators in overweight/obese patients [36]. We suggested that the low energy and nutrient intake on athletes may be associated with low levels of IL-6 at rest. Unexpectedly, we did not observe a correlation on nutrient intake and IL-6 after marathon. Recently, Badenhorst [37] also suggested an ineffective role of higher carbohydrate ingestion on IL-6 levels seven days after exercise [37]. The consumption during endurance exercise (exogenous carbohydrate) seems to be effective to modulate IL-6 levels [9] but not daily intake. The role of other macro- and micronutrient intake on IL-6 levels should be investigated in athletes.

## 5. Conclusions

Nutritional strategies to promote adequate and health diet for amateur runners may avoid relative energy deficiency, dehydration, overtraining, and immune dysfunction and should be assessed and suitable before choosing immunomodulatory supplements. In this study, we confirm the importance of adequate food intake in amateur athletes and we highlight the importance of daily energy intake (>45 kcal·kg^−1^ FFM·day^−1^), carbohydrate (8-12 g/kg), fiber (>25 g), calcium (1000 mg), and electrolytes (1.7 to 2.9 g of salt during a prolonged exercise bout), to inflammatory process induced by endurance exercise in the precompetition period.

## Figures and Tables

**Table 1:** Daily energy intake, macronutrient, cholesterol, and fiber intake.

	Daily intake	DV^∗^
Energy intake (kcal)	2258 ± 125	2908 ± 40
Carbohydrate (g/kg)	3.9 ± 0.3	^∗^8-12
Protein (g/kg)	1.6 ± 0.1	^∗^1.2-1.7
Total fat (% of EI)	30 ± 1	<30%
SFA (g)	24.6 ± 7	**—**
MUFA (g)	26.7 ± 4	**—**
PUFA (g)	16 ± 4	**—**
Sucrose (% of EI)	7 ± 1	<10%
Cholesterol (mg)	305.4 ± 160	<300
Fiber (g)	21 ± 2	>25

^∗^Reference daily values (DV) based on Dietary Reference Intake (DRI) or American Dietetic Association, Dietitians of Canada, and the American College of Sports Medicine (ADA/ACSM). SFA: saturated fatty acids, MUFA: monounsaturated fatty acids, PUFA: polyunsaturated fatty acids. The values are presented of mean ± error mean standard of 44 runners.

**Table 2:** Micronutrient daily intake in marathon runners.

Vitamins	Daily intake	DV^∗^	Minerals	Daily intake	DV^∗^
Vitamin A (mcg)	1061 ± 162	900	Calcium (mg)	656 ± 53	1000
Vitamin B1 (mg)	1.71 ± 0.1	1.2	Iron (mg)	15.5 ± 1.4	8
Vitamin B2 (mg)	1.75 ± 0.15	1.3	Mn (mg)	2.53 ± 0.24	2.3
Vitamin B3 (mg)	29 ± 3	16	Se (mcg)	169 ± 21	55
Vitamin B6 (mg)	2.4 ± 0.3	1.7	Zinc (mg)	12.2 ± 0.9	11
Folic acid (mg)	275 ± 32	400	Mg (mg)	280 ± 20	420
Vitamin B12 (mcg)	4.3 ± 0.38	2.4	P (mg)	1319 ± 79	700
Vitamin C (mg)	144 ± 37	90	Potassium (g)	2.7 ± 1.6	4.7
Vitamin D (mcg)	3.00 ± 0.53	15	Sodium (g)	2.6 ± 1.6	1.5
Vitamin E (mg)	14 ± 1.5	15			

^∗^Reference daily values (DV) based on Dietary Reference Intake (DRI) or American Dietetic Association, Dietitians of Canada, and the American College of Sports Medicine (ADA/ACSM). Mn: manganese, Se: selenium, Mg: magnesium, P: phosphorus. The values are presented as mean ± error mean standard of 44 runners.

**Table 3:** Correlation of daily intake with interleukin-6 (IL-6) before the race and interleukin-8 (IL-8) after the race in marathon runners.

Daily intake	*p* value	*r*	Daily intake	*p* value	*r*
Correlation of IL-6					
EI (kcal)	0.010	-0.39	Vitamin B2 (mg)	0.047	-0.30
Carbohydrate (g)	0.056	-0.29	Calcium (mg)	0.032	-0.32
Protein (g)	0.009	-0.39	Phosphorus (mg)	0.013	-0.37
Fat (g)	0.003	-0.43	Zinc (mg)	0.029	-0.33
Vitamin A (mg)	0.016	-0.36	Potassium (g)	0.032	-0.32
Correlation of IL-8					
EI (kcal)	0.013	-0.37	Iron (mg)	0.042	-0.31
Carbohydrate (g)	0.002	-0.45	Calcium (mg)	0.008	-0.39
Fiber (g)	0.001	-0.51	Potassium (g)	0.024	-0.34
Fat (g)	0.038	-0.31	Sodium (mg)	0.008	-0.39

EI: daily energy intake. The values are presented as *p* value and correlation coefficient (*r*) of 44 runners.

## Data Availability

The data used to support the findings of this study are included within the article.
